# Ionic Conductivity and Cycling Stability Improvement of PVDF/Nano-Clay Using PVP as Polymer Electrolyte Membranes for LiFePO_4_ Batteries

**DOI:** 10.3390/membranes8030036

**Published:** 2018-07-01

**Authors:** Endah R. Dyartanti, Agus Purwanto, I. Nyoman Widiasa, Heru Susanto

**Affiliations:** 1 Department of Chemical Engineering, Diponegoro University, Semarang 50275, Indonesia; endahrd@gmail.com (E.R.D.); widiasa@undip.ac.id (I.N.W.); 2 Department of Chemical Engineering, Universitas Sebelas Maret, Surakarta 57126, Indonesia; aguspur@uns.ac.id; 3 Membrane Research Center, Diponegoro University, Semarang 50275, Indonesia

**Keywords:** polymer electrolyte membranes, nano-clay, poly(vinylpyrrolidone), PVDF membranes

## Abstract

In this paper, we present the characteristics and performance of polymer electrolyte membranes (PEMs) based on poly(vinylidene fluoride) (PVDF). The membranes were prepared via a phase-inversion method (non-solvent-induced phase separation (NIPS)). As separators for lithium battery systems, additive modified montmorillonite (MMT) nano-clay served as a filler and poly(vinylpyrrolidone) (PVP) was used as a pore-forming agent. The membranes modified with an additive (8 wt % nano-clay and 7 wt % PVP) showed an increased porosity (87%) and an uptake of a large amount of electrolyte (801.69%), which generated a high level of ionic conductivity (5.61 mS cm^−1^) at room temperature. A graphite/PEMs/LiFePO_4_ coin cell CR2032 showed excellent stability in cycling performance (average discharge capacity 127 mA h g^−1^). Based on these results, PEMs are promising materials to be used in Polymer Electrolyte Membranes in lithium-ion batteries.

## 1. Introduction

Energy storage devices are in high demand for applications in renewable energy, transportation, and mobile electronic devices. Secondary batteries (lithium-ion) are the current state-of-the-art in energy storage devices because they offer high-energy density, they are safe for the environment, and they provide a long life-cycle [1,2]. However, common lithium-ion batteries (LIBs) that use a liquid electrolyte have some weaknesses, i.e., security problems, inflexibility, and a lack of interfacial compatibility with electrodes [3]. In the current LIB industry, a microporous polypropylene membrane is used as a separator. This separator avoids physical contact between a cathode and an anode. However, this separator must also have the ability to serve ionic transfers between the electrodes [4,5]. To be used as a battery, a polymer electrolyte must be sandwiched between battery electrodes. The presence of liquid electrolyte in a battery causes security problems due to leakage. To overcome the leakage problem, polymer electrolyte membranes (PEMs) were introduced with a solid polymer electrolyte and liquid electrolyte [6,7]. The performance of PEMs depends on the entrapment of liquid electrolytes in the polymer host matrix.

Lee et al. [8] reported four types of electrolyte membranes, namely ceramic electrolyte membranes, polymer electrolyte membranes, polymer gel electrolyte membranes and composite electrolyte membranes. Among the four types of electrolyte membranes, the polymer electrolyte membrane has the highest level of ionic conductivity. This type of electrolyte also has the advantage of high thermal stability, wide electrochemical window, and has good compatibility with the electrode. Sekhon et al. [9] reported the use of an electrolyte membrane composed of a poly(vinylidene fluoride) (PVDF)-based polymer gel containing a ternary solvent of ethylene carbonate, propylene carbonate and dimethyl acetamide. They achieved a relatively high ionic conductivity of about 10^−4^ S cm^−1^ at 20 °C. Furthermore, Choi et al. [10] reported the use of a PAN-based electrolyte membrane to obtain an electrolyte membrane with an ionic conductivity of about 10^−3^ S cm^−1^ and a stable electrochemical window of up to 4.5 V. Saunier et al. [11] fabricated a PVDF-based electrolyte membrane using liquid electrolyte (1 M LiPF_6_ in EC, DMC, DEC). The electrolytic membrane produced by this higher electrolytic ionic conductivity increased by increasing the porosity of the micropore membrane. Some researchers have reported PVDF-based polymer gel electrolytes produced by electrospinning [12] that have electrolyte conductivities about 10^−3^ S cm^−1^.

The polymer types that have been used as polymer matrices for PEMs include poly(vinylidene fluoride) (PVDF) [9], poly(methyl methacrylate) (PMMA) [10], and polyacrylonitrile (PAN) [11]. Among them, PVDF is the most widely used because it has a high dielectric constant, high affinity for a liquid electrolyte, and relatively high dimensional stability compared with PVDF-HFP [12]. Thus far, the use of PVDF as PEMs has presented two main problems: PVDF is partially soluble in liquid electrolytes [13], and it has a high level of crystallinity that lowers ion conductivity [14]. To improve the ion conductivity and ion mobility, fillers such as Al_2_O_3_, SiO_2_, BaTiO_3_, and nano-clay were added to the polymer matrix [3,13,15,16,17,18]. The fillers also enhanced the Lewis acid-base interaction between a separator and an electrolyte, which increases the number of sites for ion transfer and minimizes the formation of ion pairs. Riley et al. described that the introduction of lithium hectorite clay into the host matrix polymer is an appropriate method to reduce the formation of ion pairs [19]. In the present investigation, nano-clay was used as a filler because of its advantages such as low cost and naturally available materials.

Clay is most commonly used as an inorganic layered filler given its unique characteristics. The clay has length scale (channel width = 16 Å), high cation exchange capacity (∼80 meq/100 g), suitable interlayer charge (∼0.55), and a substantial specific surface area (∼31.82 m^2^ g^−1^) [20]. A large specific surface area provides excellent mechanical properties of the polymer matrix and dielectric property [21]. Riley et al. [19] described how the addition of nano-clay into the matrix of polymer would minimize ion-pair formation, which can reduce the amount of mobile charge carriers that can cause low ionic conductivity in PEMs. The clay function in PEMs directly avoids anion counter mobility [22]. The cationic charges on the top of a clay particle play a role as Lewis acid centers and construct complexes with the polymer. This tailors the pore structure and dissociation of the lithium salt. The membrane electrolytes with nano clay filler exhibit excellent thermal stability, barrier characteristic, dimensional stability, and chemical resistance [23].

To improve the conductivity of ion in a membrane, a highly porous membrane must guarantee the maximum loading of an electrolyte. A polymer electrolyte membrane should have interconnected micropores that are adequately distributed. The addition of a pore-forming agent in a host polymer matrix will improve the structure of the polymer electrolyte membrane, as prepared in previous publications [24,25,26,27]. PVP is a potential pore-forming agent used in PVDF membranes synthesized via the phase inversion method [12,24,25,26]. A few studies have reported evidence showing that pore-forming agents such as urea [27,28] polyethylene glycol (PEG) [21,29], salicylic acid [30], and polyvinylpyrrolidone (PVP) [24,25,26] can enhance ionic conductivity via an increase in the porosity of membranes.

Further, incorporating pore-forming agents and filler in the preparation of polymer electrolyte membranes reportedly increases battery performance. Li and co-workers explored the co-effects of urea and SiO_2_ and found electrochemical stability, mechanical strength, and improvements in the ionic conductivity, interfacial properties, and in the transference number of polymer electrolytes [28]. Meanwhile, Liu et al. [12] have reported the influence of graphene on the improvement of conductivity and the electrolyte uptake of PVDF-based polymer electrolytes when using PVP as a pore-forming agent. The PEMs showed the increase in the ionic conductivity and higher specific discharge capacity. These studies were focused on uniformly dispersed graphene in a polymer matrix. However, the effect of pore-forming agents (PVPs) has not been investigated.

In this paper, we report the ionic conductivity and cycling stability improvement of PVDF/nanoclay as Polymer Electrolyte Membranes for LiFePO_4_ Batteries. This research is important because the coactions of PVP and nano-clay significantly improved the performance of the polymer electrolytes in term of the electroactive phase, the degree of crystallinity, porosity, electrolyte uptake, the C-rate discharge capacity and the ionic conductivity. To the best of our knowledge, the literature cites no reports of nano-clay PVDF PEMs prepared using PVP as pore-forming agents. These nano-clay PVDF PEMs were structurally modified by PVP and showed high ionic conductivity and stable cyclability.

## 2. Results and Discussion

### 2.1. Morphology Characterization

**Figure 1:**
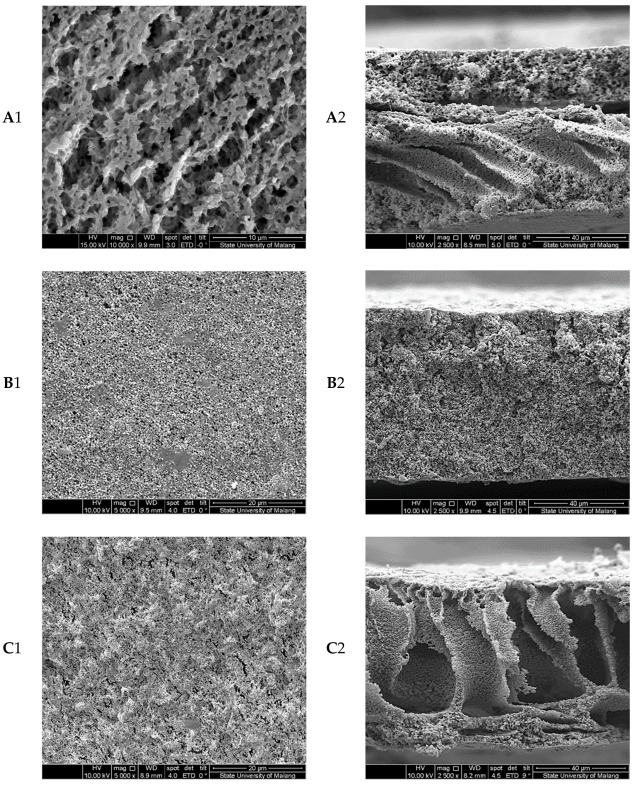

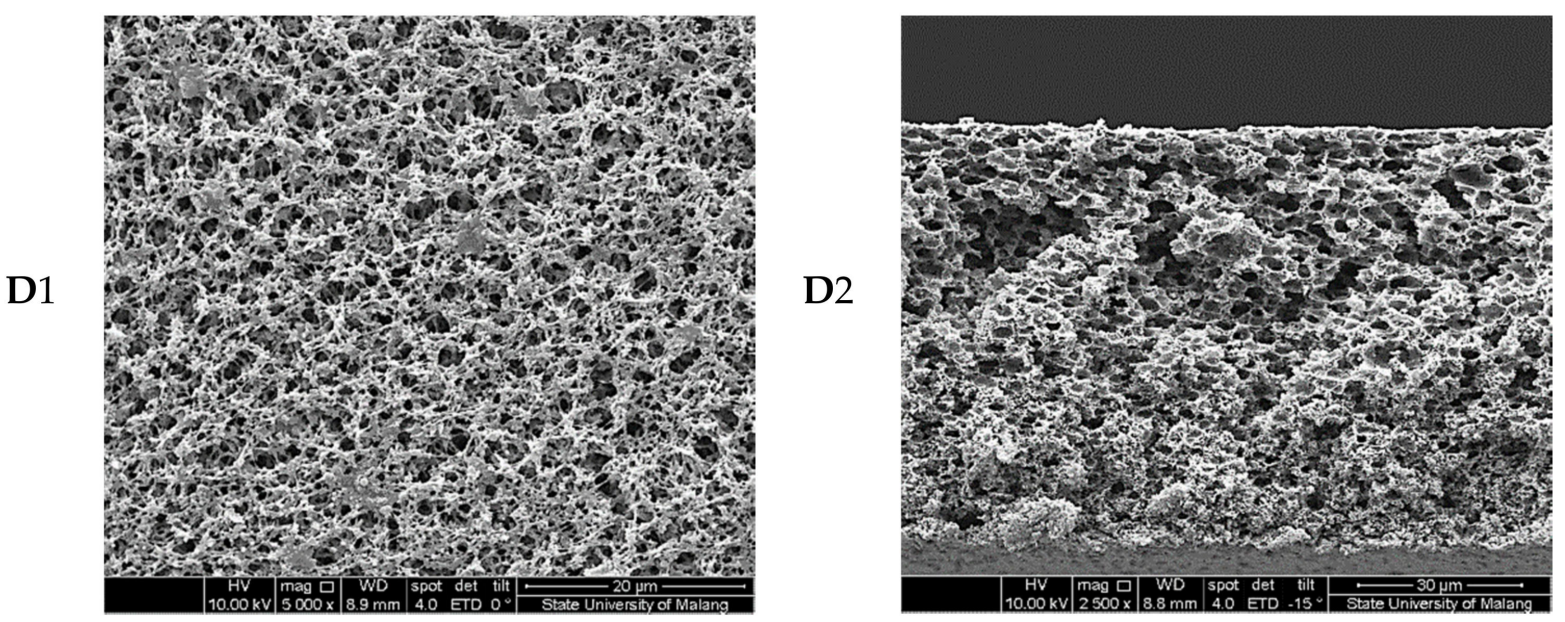
Top view (1) and Cross section (2) view of polymer electrolyte membranes (PEMs): (**A**) poly(vinylidene fluoride) (PVDF) (10 wt %); (**B**) PVDF (10 wt %) with nano-clay (8 wt %); (**C**) PVDF (10 wt %) with PVP (7 wt %); and (**D**) PVDF (10 wt %), with polyvinylpyrrolidone (PVP) (7 wt %) and nano-clay (8 wt %).

Many previous publications have reported that PEM performance is greatly influenced by the membrane structure [31,32,33]. Further, the membrane structure is influenced by factors such as the solvent-nonsolvent system, pore-forming agents, fillers, preparation conditions, and polymer concentration [29,34,35,36]. In this work, the co-effect of a pore-forming agent (PVP) and filler (nano-clay) exerted on the structure of PEMs was investigated. The results are shown in Figure 1.

In Figure 1, B1 and B2 showed the PVDF with filler additive nano-clay membrane were more porous than the surface of pure PVDF membranes. The membrane structure was the result of preferential phase inversion caused by the introduction of the clay filler. Intercalation of nano-clay into PVDF polymer using non-solvent induced phase separation (NIPS) method is based on the dimethylacetamide (DMAc) solvent system. When the modified nano-clay particles are dispersed in polymer solution, the polymer chains intercalate and displace the solvent within the interlayer of the clay. Addition of 8 wt % nano-clay in the PVDF polymer provided uniform pores structure. This structure of membrane pores could produce a higher porosity for greater uptake of electrolytes [37].

The membrane-based PVDF with PVP additives showed a significantly higher porosity and pore size (Figure 1(C1,C2)) compared with membranes with no additive. In Figure 1(C1,C2), a section of similar columnar macro voids or finger-like structures seemed in the dominant section of the membrane cross-section. Note that only 7 wt % of PVP was mixed in membrane solution in Figure 1(C1,C2). It is principal to note that some voids looked under the top layer by adding the PVP. The macro voids may be built from PVP leaving areas. The PVP is an additive that functions in forming the pores and voids in the membrane [18]. This can be explained by the thermodynamic effect of PVP. The demixing polymer solution by means of nucleation and growth of a rich polymer phase will greatly influence the formation of pore membranes structure. The addition of PVP in the casting solution serves as a nonsolvent for PVDF polymer, which will cause the polymer solution to have lower thermodynamic stability thereby increasing the phase separation. Therefore, the increasing demixing rate of the interface due to the addition of PVP will lead to rapid collapse of the polymer chain and the formation of a larger gap between collapsed chains [38]. The result of membrane modification showed that the addition of PVP significantly reduced the thermodynamic stability of the casting solution.

The PVDF/PVP/nano-clay membranes showed the highly interconnected sponge-like porous structure (Figure 1(D1,D2)), which ensures their dimension stability. The unique microporous structure is formed during the solvent and non-solvent exchange process in the mixing coagulation bath [16]. Moreover, as shown in Figure 1(D2), the top view of modified membranes with PVP and nano-clay gets a much more uniform morphology and more compacted structure compared to modified PVDF membranes with no additive. The pore size and the shape formation in PVDF/PVP/nano-clay membranes depend on the three factors including the delay of liquid-liquid phase separation process, the polymer-based matrix and the additive [39].

### 2.2. Surface Chemistry

**Figure 2:**
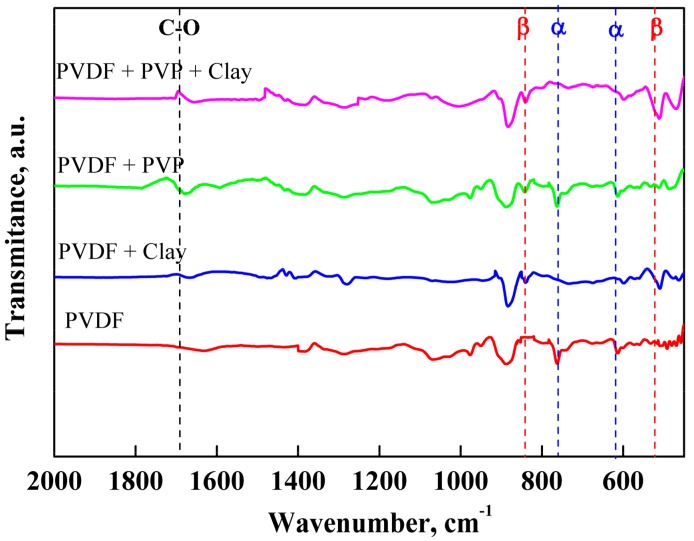
FTIR spectra of pure PVDF membranes and modified PEMs with nano-clay, PEMs with PVP, and PEMs with nano-clay and PVP.

**Figure 3:**
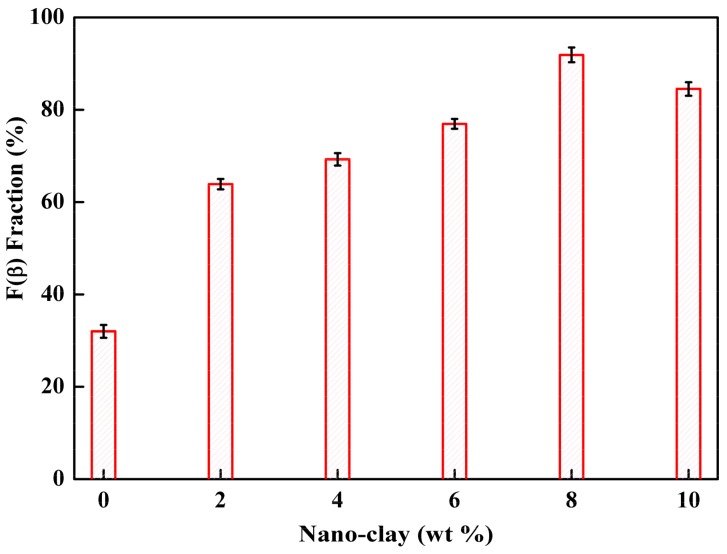
The β-phase fraction F_(β)_ of membranes with different contents of Nano-clay particles.

The surface chemistry of PEMs was characterized using FTIR. The FTIR studies of PEMs were carried out to determine the appearance of the β-PVDF. The FTIR spectra of pure PVDF and modified PEMs are shown in Figure 2. With pure PVDF, the characteristic peaks at 1414 cm^−1^, 1233 cm^−1^, 1176 cm^−1^ and 881 cm^−1^ were assigned to –CH_2_– deformation [40], –C–F– stretching, and –CF_2_– stretching, and an amorphous band of PVDF was noted [41]. Characteristic bands of the α-phase were observed at 763 (–CH_2_– rocking) and 615 cm^−1^ (–CF_2_– bending and CCC skeletal bending). The characteristic peaks of β-PVDF were observed at 840 and 510 cm^−1^. Furthermore, the bands at 840 cm^−1^ indicated –CH_2_– rocking and –CF_2_– stretching, and those at 510 cm^−1^ indicated CF_2_. It is noteworthy that PVDF/nano-clay/PVP PEMs showed one peak at 1661 cm^−1^ (C–O) from the combined contribution of nano-clay and carbonyl groups of PVP. The fraction of the electro-active β-phase, F(β), in PEMs was calculated using the Lambert-Beer law stated in Equation (1). The relationship between the F(β) with clay and the PVP content (% weight) is depicted in Figure 3. The β-phase fraction increased with increasing the concentration of nano-clay. However, beyond a certain concentration of nano-clay, the β-phase fraction decreased. The highest β fraction (91%) was obtained with a concentration of nano-clay at 8%. The β phase of PVDF presented the highest electroactive properties. The addition of modified montmorillonite to PVDF promoted the change of α to β phases of the PVDF crystals [42]. The degree of their dielectric properties was equivalent to the number of the β-phase in the PEMs. Therefore, it can be verified from the FTIR data that the nano-clay increased the nucleation as well as the stabilization of the electroactive β phase in PVDF.

### 2.3. Characterization of Crystallinity

Thermal analysis and identification of the crystalline phases of PEMs were performed using differential scanning calorimetry (DSC) and thermogravimetric analysis. In this section, the effects of nano-clay and PVP on thermal properties and the crystallinity of PEMs was investigated. The results are presented in Table 1. The membranes exhibited a change in the melting point (T_m_) with increasing contents of nano-clay, but this also caused small variations in the heat of fusion (ΔHm). The degree of crystallinity (Χ_C_) for PEMs was calculated from the DSC curve based on Equation (4).

The crystallinity of modified PEMs ranged from 14.71–27.71%. The modified PEMs with 8 wt % nano-clay and 7 wt % PVP exhibited a small ΔHm (15.3869 J/g) and Xc (14.71%), which was attributed to high amorphicity. The ionic conductivity of modified membranes in the amorphous phase was higher compared with the crystalline phase. The polymer chains in this phase were more flexible and led to increased segmental displacement in the membranes.

In this study, wet PVDF/PVP/nano-clay membranes were prepared by the immersion of the PVDF/PVP/nano-clay membranes in LiPF_6_ solution for 2 h, the electrolyte solution penetrates the pores of the membranes, occupies the pore space, and swells the polymer chain. At the same time, nano-clay acts as a filler in the PVDF/PVP system in which there is an interaction between nano-clay, PVP and PVDF. The LiPF_6_ solution uptake of the PVDF/PVP/nano-clay membranes increased with increasing nano-clay content up to 8 wt % but then decreased with further increasing nano-clay content up to 10 wt %. The LiPF6 solution uptake is not directly correlated with porosity. This result was attributed to not only the porous structure but also to the swelling movement of PVDF polymer chains and the possible hydrophilicity of the nano-clay surface groups. Therefore, membranes with high amorphicity resulted in higher ion conductivity. These results were lower than PVDF PEM membranes prepared using graphene/PVP as a pore-forming agent (44%) [12].

### 2.4. Porosity and Electrolyte Uptake

**Figure 4:**
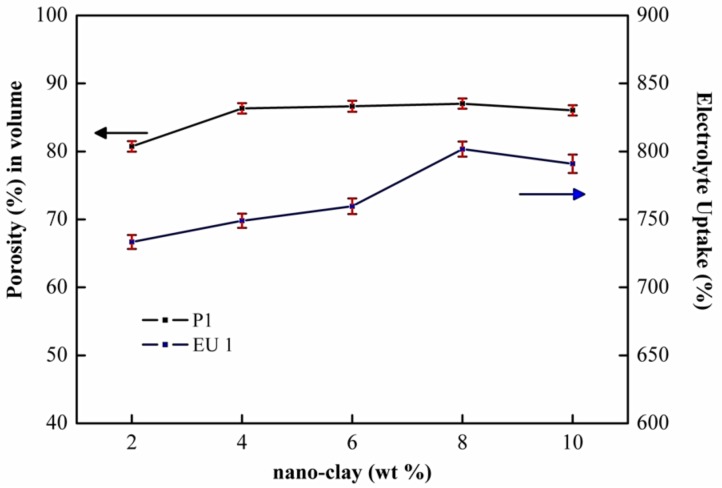
Porosity (%) in volume and Electrolyte uptake (%) of the PVDF/PVP/nano clay membranes with different levels of clay loading at PVP 7 wt %.

The PEMs were immersed in a solution of n-butanol, and Equation (2) was applied to determine the porosity of the PEMs. The effect of nano-clay content on the membranes porosity and electrolyte uptake of PEMs is shown in Figure 4. The highest porosity of PEMs, 87%, was obtained via the addition of 8 wt % nano-clay and 7 wt % PVP. This porosity was higher than that of PEM composed of PVDF (75%), as reported by Deka and Kumar [43], and is comparable to PEM made of PVDF with the addition of PVP and graphene (88%), as reported by Liu [13]. These results confirmed the performance of the addition of PVP.

The porosity and pore structure of membranes are essential factors in the electrolyte uptake of a PEM. The electrolyte uptake was calculated by dipping the membranes in a liquid electrolyte for 2 h. The electrolyte uptake was calculated using Equation (3). The highest electrolyte uptake (801.69%) was demonstrated by PVDF PEMs with additions of 8 wt % nano-clay and 7 wt % PVP. As shown in Figure 4, the electrolyte uptake of PEMs (4% nano-clay and 7% PVP) was 749%. It was much higher than the electrolyte uptake of PVDF PEMs with additions 4% nano-clay, which was 177%, as reported by Deka and Kumar [43]. Prasanth et al. [44] reported the highest electrolyte uptake of PVDF membranes with surface modification of clay at 246% for 1 wt % clay addition. With a more porous structure, PEMs are expected to absorb more liquid electrolyte. It was proven by the data from electrolyte uptake.

The PEMs had a higher level of electrolyte uptake (EU) than the PVDF membranes; the values for EU of pure PVDF, PVDF/nano-clay, and PVDF/nano-clay/PVP membranes were 326.30, 354.50, 577.80 and 801.6%, respectively. That results agreed with the SEM and porosity analysis shown in Figure 1, wherein higher pore size and distribution density resulted in higher levels of liquid uptake.

### 2.5. Ionic Conductivity

One of the crucial characteristics of PEM for LIB separator applications is ionic conductivity. In this work, the effect of nano-clay and PVP on the ionic conductivity of PEMs was investigated. The ionic conductivity was determined by sandwiching SS/Polymer electrolytes/SS (stainless steel (SS) blocking electrodes) cells at room temperature.

**Figure 5:**
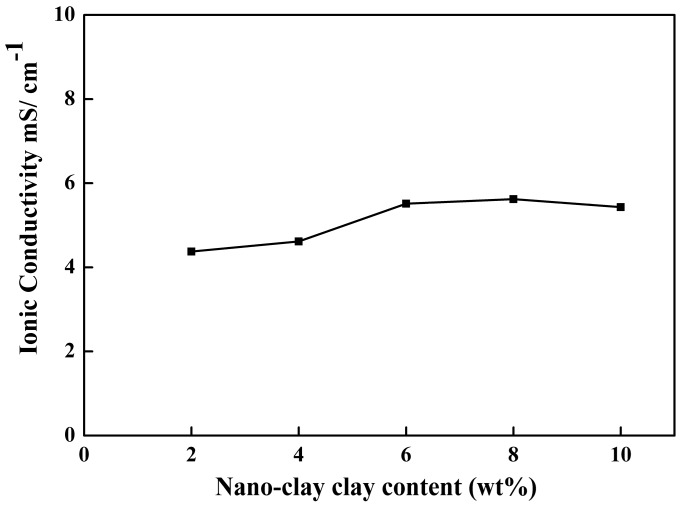
The Ionic Conductivity of PVDF membranes with different contents of clay at 7 wt % PVP.

The results in Figure 5 show that the maximum ionic conductivity, about 5.61 mS cm^−1^, was achieved by a PVDF PEM membrane prepared with the addition of 8 wt % nano-clay and 7 wt % PVP. Excessive addition of filler in the PEMs promoted aggregation that decreased the volume of the interface layer.

Several studies as shown in Table 2, have reported the effect of fillers and pore-forming agents on increasing the ionic conductivity. The introduction of a nano-clay filler into the polymer PVDF with PVP as a pore-forming agent in those studies, however, has shown ionic conductivity that is higher than that of the PEMs with SiO_2_-urea and grapheme-PVP. Increased porosity and reduced the degree of crystallinity in the polymer will enhance the ionic conductivity of polymer electrolytes. High-porosity PEMs can store a more significant number of electrolytes and delivers more channels for ion migration. The enhance of ionic conductivity of the PEMS electrolyte ions is also due to the placement of nano-clay on the internal surface of the pore channel. This can create a lithium ion conduction pathway through Lewis acid-base interactions or around them [23]. Furthermore, the oxygen-containing functional groups in nano-clay would increase the migration of lithium ions.

### 2.6. Rate Performance

**Figure 6:**
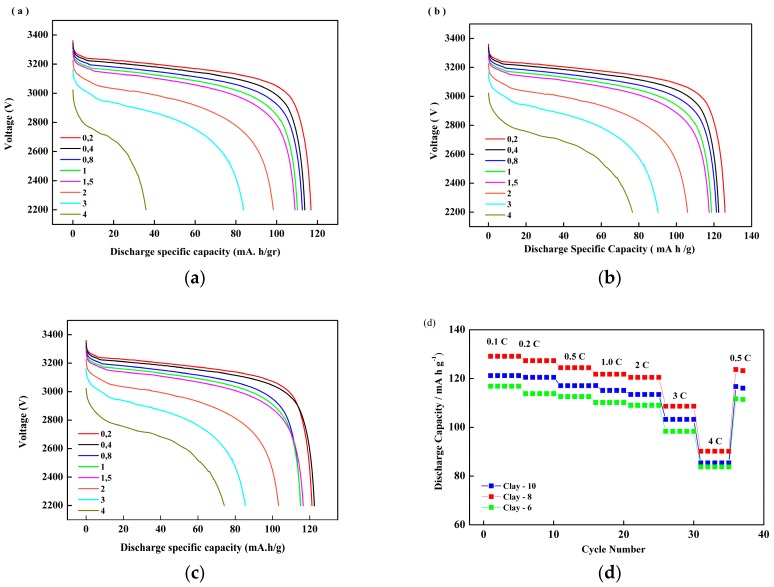
Discharge profiles of LiFePO_4_/PEMs/graphite cells constructed with different nano-clay loading and different discharge capacity rates: (**a**) 6 (wt % PVDF); (**b**) 8 (wt % PVDF); (**c**) 10 (wt % PVDF); (**d**) Discharge specific capacity of battery test with different nano-clay loading (clay 8–10) and different discharge rate (0.1–4 C) (in the voltage range 2.2–3.65 V).

The cycling performance of battery cells was analyzed using LiFePO_4_ as a cathode, graphite as an anode, and as-prepared membranes. Figure 6 and Table 3 show representative discharge/charge voltage profiles of LiFePO_4_/PEMs/graphite battery testing at a current rate of 0.1 C (1 C = 2 mA), in the range of voltage 2.2–3.65 V, under ambient temperature for the first cycle. Charge and discharge rates of a battery are governed by C-rates. The capacity of a battery at 1 C means that a fully charged battery rated for one hour. The battery discharging at 0.2 C should provide 5 h. After the preconditioning cycle, the cell was charged at a current-rate of 0.2 C (0.4 mA) to a target voltage of 3.65 V. This was followed by a constant–voltage charge with a charging current rate of 0.3 mA. It was discharged at a cutoff voltage of 2.2 V and a current density of 0.4 mA.

As shown in Table 3, the discharge capacities of the PEMs with 8 wt % nano-clay concentration (Figure 6b) were superior to PEMs with 6 wt % (Figure 6a) and 10 wt % (Figure 6c). The difference in the discharge capacity became even larger at higher discharge current densities for different nano-clay filler concentrations. The better electrochemical performance membranes in LiFePO_4_/PEMs/graphite batteries may be ascribed to high porosity, and electrolyte uptake, as shown in Figure 4. The interfacial compatibility between the additives and the PEMs increased the ionic conductivity [23,24,25].

**Figure 7:**
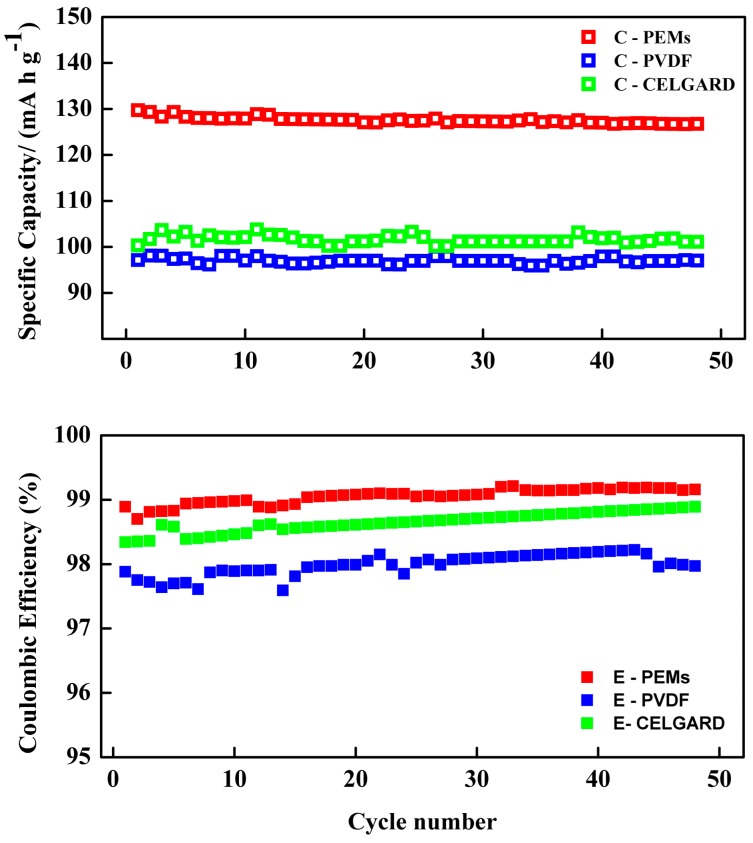
Discharge capacity and Coulombic Efficiency for the graphite/PEM/LiFePO_4_ cells at a C rate of 0.2 C (0.4 mA).

Figure 7 shows discharge capacity and coulombic efficiency (ratio of the discharge capacity divided by the charge capacity) as functions of the cycle number for the 1st, 10th, 20th and 48th cycles of a LiFePO_4_/PEMs/graphite batteries with different separators. The initial discharge capacities of as-prepared PEMs, Celgard, and PVDF separators were 129.733, 100.3 and 97.119 mA h g^−1^, respectively. After 48 cycles, the final discharge capacities were 126.765, 101.126 and 96.915 mA h g^−1^. The fade-in capacities per cycle of PEMs, Celgard, and pure PVDF were 0.06, 0.004 and 0.01 mA h g^−1^, respectively. The average discharge capacity after 48 cycles of the modified PEMs (127 mA h g^−1^) was higher than either the Celgard separator (101 mA h g^−1^) or pure PVDF (96.99 mA h g^−1^), by comparison with the theoretical capacity of LiFePO_4_ at 170 mA h g^−1^. The decrease in the capacity was thought to be due to the degradation of the electrode/polymer interface, which gradually raises the internal resistance in the battery during the charge/discharge cycles. This behavior was also reported by Liu et al. [12]. The PVDF/graphene gel electrolyte in LiCoO_2_/Li cells had a discharge capacity of 149 mA h g^−1^ with a C rate of 1 for LiCoO_2_ and a theoretical capacity as high as 273.8 mA h g^−1^.

As shown in Figure 7, the coulombic efficiency steadily increased with cycle number. It is clear that the separator modified by PVP and nano-clay had a higher coulombic efficiency than pure PVDF with a commercial Celgard separator. A coulombic efficiency of less than 100% is probably associated with the oxidation of the solvent. Compared with pure PVDF and Celgard (commercial separator), the best discharge capacity of the PEMs was associated with the structure of the pores, higher absorption of liquid electrolyte, ionic conductivity, and good compatibility of the cathode and anode.

## 3. Materials and Methods

### 3.1. Materials

This research used poly(vinylidene fluoride) (PVDF) (Sigma-Aldrich, MW 534000, Saint Louis, MO, USA) as the polymer matrix. The pore-forming agent was polyvinylpyrrolidone (PVP) (purchased from Merck, MW 25,000 g/mol). *n*,*n*-dimethylacetamide (DMAc) was purchased from Merck and used directly as a solvent without further purification. We also used Nano-clay (Nanomer 1.31PS) produced by Nanocor, Inc. (Chapel Hill, NC, USA) (MMT, Sigma-Aldrich); modified montmorillonite with octadecylamine (15–35%) and aminopropyltriethoxysilane (0.5–5%); and, 1 M of lithium hexafluorophosphate (LiPF_6_) in a mixture of ethylene carbonate (EC)/dimethyl carbonate (DMC)/diethyl carbonate (DEC) (4:2:4 by volume) (MTI Corporation, Richmond, CA, USA).

### 3.2. Membrane Preparation

First, nano-clay particles were dispersed in a DMAc solvent using an ultrasonic sonicator at a temperature of 35 °C. After that, a certain concentration (% weight.) of PVDF was added under continuous stirring for 3 h at 45 °C. Then, a certain concentration of PVP was mixed with the solution and stirred for at least 2 h to achieve a homogenous solution. The solution mixture was let stand without stirring until all bubbles had disappeared. The solution mixture was cast on a glass support with a thickness of 150 μm. The casting process was conducted with a Doctor Blade coating machine (MSK-AFA-II, MTI corp.). The as-prepared membranes were directly immersed in nonsolvent (deionized water) at 25 °C for 72 h. Before drying in a vacuum oven, the formed membranes were desiccated under air at 25 °C for 24 h. Table 1 shows the various membrane compositions prepared in this study. In this work, the membranes were prepared at ambient condition (temperature 26 + 2 °C and relative humidity 40 + 5%).

### 3.3. Membrane Characterization

The pore structure of nano-clay/PVDF PEMs was examined with a digital scanning electron microscope (FEI Inspect S50, SEM) after gold-palladium (180 s) was sprayed onto the surface of the membranes. To observe cross-sectional views of the membranes, the samples were immersed in liquid nitrogen. The spectra of membrane electrolytes were obtained using Fourier Transform Infrared Spectroscopy (FTIR) (Shimadzu Prestige-21). The β-phase F(β) fraction in the membranes was calculated from the FT-IR spectra using the Lambert-Beer law given in ref. [45].

(1) $$F\left(\beta \right)\;=\;\frac{A_{\alpha }}{\left(\frac{K_{\beta }}{K_{\alpha }}\right){\;A}_{\alpha }\;+{\;A}_{\beta }}$$

In Equation (1) A_α_ (764 cm^−1^) and A_®_ (840 cm^−1^) are absorbance values, and K_α_ and K_β_ are the absorption coefficients of (6.1 × 10^4^ cm^2^ mol^−1^) and (7.7 × 10^4^ cm^2^ mol^−1^), respectively, at their respective wavenumbers.

Membrane porosity (P) was measured by an absorption method with the n-butanol solution. The use of n-butanol followed previous publication by [16,44,46]. In this method, the membrane was first weighed, then soaked in the n-butanol solution for 2 h After that, the wet membrane was weighed, and the mass of the n-butanol absorption was calculated. The membrane porosity (P) % in volume was calculated using Equation (2).

(2) $$P\;(\%)\;=\;\frac{M_{\mathrm{BuOH}\;}{/\rho }_{\mathrm{BuOH}}}{M_{\mathrm{BuOH}\;}{/\rho }_{\mathrm{BuOH}}{\;+\;M}_{p}{/\rho }_{P}}\;\times \;100$$

In Equation (2), M_p_ and M_BuOH_ represent the dry and wet membranes with butanol mass, ρ_P_ is the membrane density, and ρ_BuOH_ is the density of the membrane following the absorption of butanol [16]. The membrane’s porosity was averaged from three measurements.

The membranes were immersed in the LiPF_6_ solution for 2 h, and then the uptake of the electrolyte solution (δ) was calculated using Equation (3).

(3) $$\delta \;\left(\%\right)\;=\;\frac{M\;-{\;M}_{0}}{M_{0}}\;\times \;100\;\%$$

In Equation (3), M_o_ and M are the mass of the dry and wet membranes, respectively. Thermal and crystalline properties of the membranes were observed by differential scanning calorimetry (Perkin Elmer DSC-7, Waltham, MA, USA) at a heating rate of 10 °C min^−1^ from 20 to 350 °C and thermogravimetric analysis (Linseis STA Platinum Series) at a heating rate of 10 °C min^−1^ from 30 to 600 °C under a N_2_ atmosphere. From the DSC data, the degree of crystallinity (X_C_) of each sample was determined using Equation (4).

(4) $$X_{C}\;\%\;=\;\frac{\Delta H_{\mathrm{sample}}^{m}}{\Delta H_{m}^{⋆}}\;\times \;100$$

In Equation (4), X_C_ is crystallinity, $\Delta H_{\mathrm{sample}}^{m}$ is the heat of fusion per gram sample, $\mathrm{and}\;\Delta H_{m}^{⋆}$ is the heat of fusion per gram of total crystalline polymer, $\Delta H_{m\;}^{⋆}\mathrm{PVDF}$ = 104.7 J g^−1^ [16].

### 3.4. Electrochemical and Battery Performance

Electrochemical evaluation as ionic conductivity analysis was performed by dipping a membrane sample (area ∼1.9 cm^2^) into a liquid electrolyte −1 M lithium hexafluorophosphate (LiPF6) in (EC (ethylene carbonate)/DMC (dimethyl carbonate)/DEC (diethyl carbonate) (4:2:4 by volume). The samples were inserted between SS blocking electrodes (16 mm) in CR2032 coin cell cases. The impedance calculations were conducted using a HIOKI LCR Hi-Tester Model 3532 for frequencies ranging from 42 Hz to 5 MHz at an amplitude of 10 mV. Moreover, the ionic conductivity was calculated by Equation (5).

(5) $$\sigma \;=\;\frac{d}{R_{b}\;S}$$

In Equation (5), σ is the ionic conductivity, *R_b_* is the bulk resistance, and *d* and *S* are the thickness and area of the specimen, respectively.

The coin cell batteries (CR2032) were sandwiched in a glove box with a moisture level lower than 10 ppm, to analyze the performance of as-prepared Polymer electrolyte membranes (PEMs) in the LiFePO_4_ battery. The gel electrolyte was sandwiched between the anode (graphite) and a cathode of lithium iron phosphate (LiFePO_4_). The batteries were placed in an automatic charge/discharge Battery Analyzer (0.02–10 mA, MTI corp.), and tested in a voltage range of 2.5 to 3.65 V at a temperature of 25 °C with a C-rate of 0.2 C.

## 4. Conclusions

Polymer electrolyte membranes based on PVDF with PVP as a pore-forming agent with nano-clay as a filler were successfully prepared and characterized. The addition of a pore-forming agent (PVP) and filler (nano-clay) significantly improved the performance of the polymer electrolytes in aspects such as porosity, electrolyte uptake, and ionic conductivity at room temperature. Visualization of membrane morphology using SEM confirmed the porous structure of the membrane. The PEMs showed a conductivity of 5.61 mS cm^−1^ at room temperature. A fabricated cell assembled with PEMs also showed excellent stability during cycling at an average discharge capacity (127 mA h g^−1^). Thus, these novel polymer electrolyte membranes are promising candidates for use in LiFePO_4_ batteries.

## Figures and Tables

**Table 1:** Thermal properties and crystallinity of PVDF/PVP/nano-clay PEMs.

Name of Sample	Composition			Heat Fusion ΔHm (J/g)	Degree of Crystallinity X_C_ (%)	Melting Point T_m_ (°C)
	PVDF	PVP	Nano-Clay			
C-2	10	7	2	38.99	37.27	164.75
C-4	10	7	4	36.79	35.12	163.98
C-6	10	7	6	25.42	24.31	163.36
C-8	10	7	8	15.39	14.71	163.20
C-10	10	7	10	22.96	27.71	163.54

**Table 2:** Ionic conductivity of Electrolyte membranes based PVDF with Different Additives.

Polymer	Additive	Electrolyte	Ionic Conductivity (mS cm^−1^)	Ref
PVDF	PVP + nano-clay	LiPF_6_ in EC/DC/DMC	5.610	
PVDF	nano-clay	LiPF_6_ in EC/DEC	3.080	[44]
PVDF	SiO_2_-urea	LiPF_6_-EC/DMC/EMC	3.652	[28]
PVDF	PVP + graphene	LiPF_6_-EC/DMC/EMC	3.610	[13]

**Table 3:** The discharge capacity of LiFePO_4_/PEMs/graphite battery test at different nano-clay loading with different current rate (0.1–4 C).

Sample	Discharge Capacity with Different Current Rate							
	0.2 C	0.4 C	0.8 C	1 C	1.5 C	2 C	3 C	4 C
10 C	116.86	113.84	112.60	110.17	109.00	98.33	83.76	74.10
8 C	125.85	122.48	121.26	118.67	117.38	90.21	90.21	76.57
6 C	121.19	122.48	115.06	115.12	116.49	103.25	85.39	74.10
